# P-1262. New Delhi Metallo-β-lactamase (NDM) by Numbers: Using the Allele to Track Distinct Microbiologic and Epidemiologic Landscapes

**DOI:** 10.1093/ofid/ofaf695.1453

**Published:** 2026-01-11

**Authors:** Autumn Lewis, Katie Barry, Sharvari Narendra, Salvador Castañeda-Barba, Thomas J X Li, Emily Snavely, Amy Mathers

**Affiliations:** University of Virginia, Charlottesville, Virginia; University of Virginia, Charlottesville, Virginia; University of Virginia, Charlottesville, Virginia; University of Virginia, Charlottesville, Virginia; University of Virginia, Charlottesville, Virginia; University of Virginia Health; University of Virginia, Charlottesville, Virginia

## Abstract

**Background:**

The New Delhi Metallo-β-lactamase (NDM) is a carbapenemase that has been found across many bacterial species and strains and carried on over 20 distinct plasmid types. To track this mobility, we sought to develop alternative genomic approaches to understand and describe emergence of NDM-producing isolates in our region.

**Methods:**

We prospectively screened all carbapenemase-producing organisms (CPOs) from peri-rectal surveillance and clinical samples at a tertiary health system in central Virginia using the NG-Test Carba-5 lateral flow assay (NG Biotech). NDM-producing isolates from January 2020 to March 2025 underwent whole-genome sequencing with Illumina and Oxford Nanopore Technologies. Hybrid assemblies were completed using Unicycler, and sequences were analyzed using our standard bioinformatic pipeline, which includes AMRFinder and PlasmidFinder.

**Results:**

In total, 41 individual NDM isolates were collected from 26 patients. NDM increased from 6% (6/107) of CPO isolates in 2020–2022 to 30% (35/115) in 2023–March 2025 (p< 0.0001 Fisher’s exact test). The most common NDM variant found was NDM-7 (22), followed by NDM-5 (13), NDM-1 (5), and a single isolate produced NDM-50. Despite *Escherichia coli* being the most commonly detected NDM-positive species (14/41), *Klebsiella pneumoniae* was the only species in which all four NDM variants were detected. NDM-7 was exclusively carried on an IncX3 plasmid, detected across 12 unique Enterobacterales species. NDM-5 was most commonly found in *E. coli* (11/13). NDM-1 was identified in *Pseudomonas aeruginosa* (3/5 isolates), with chromosomal integration in two patients with international exposure. Most patients with NDM-1 (3/4) or NDM-5 (6/7) had international travel history, while none of the 15 patients with NDM-7 reported travel outside the U.S. (p< 0.0001).

**Conclusion:**

As NDM emerges in the mid-Atlantic region, its epidemiology and microbiologic mobility appear linked to specific alleles. A highly mobile IncX3 plasmid carrying NDM-7 is circulating locally, while other alleles (e.g., NDM-1, NDM-5) are associated with international exposure and importation. Tracking the NDM allele may provide a valuable tool for understanding the evolving epidemiology and spread of NDM in the United States.

**Disclosures:**

All Authors: No reported disclosures

